# Surveillance of Zoonotic Infectious Disease Transmitted by Small Companion Animals

**DOI:** 10.3201/eid1812.120664

**Published:** 2012-12

**Authors:** Michael J. Day, Edward Breitschwerdt, Sarah Cleaveland, Umesh Karkare, Chand Khanna, Jolle Kirpensteijn, Thijs Kuiken, Michael R. Lappin, Jennifer McQuiston, Elizabeth Mumford, Tanya Myers, Clarisa B. Palatnik-de-Sousa, Carol Rubin, Gregg Takashima, Alex Thiermann

**Affiliations:** Author affiliations: University of Bristol, Langford, UK (M.J. Day);; North Carolina State University, Raleigh, North Carolina, USA (E. Breitschwerdt);; University of Glasgow, Glasgow, UK (S. Cleaveland);; Mumbai, India (U. Karkare);; National Cancer Institute, Bethesda, Maryland, USA (C. Khanna);; Utrecht University, Utrecht, the Netherlands (J. Kirpensteijn);; Erasmus Medical Centre, Rotterdam, the Netherlands (T. Kuiken);; Colorado State University, Fort Collins, Colorado, USA (M.R. Lappin);; Centers for Disease Control and Prevention, Atlanta, Georgia, USA (J. McQuiston, T. Myers, C. Rubin);; World Health Organization, Geneva, Switzerland (E. Mumford);; Federal University of Rio de Janeiro, Rio de Janeiro, Brazil (C.B. Palatnik-de-Sousa);; Portland, Oregon, USA (G. Takashima);; World Organisation for Animal Health, Paris, France (A. Thiermann)

**Keywords:** dogs, cats, small companion animals, emerging zoonoses, zoonoses, zoonotic infectious disease, transmission, surveillance, One Health

## Abstract

The One Health paradigm for global health recognizes that most new human infectious diseases will emerge from animal reservoirs. Little consideration has been given to the known and potential zoonotic infectious diseases of small companion animals. Cats and dogs closely share the domestic environment with humans and have the potential to act as sources and sentinels of a wide spectrum of zoonotic infections. This report highlights the lack of a coordinated global surveillance scheme that monitors disease in these species and makes a case for the necessity of developing a strategy to implement such surveillance.

Increasingly, the concept of One Health is recognized as a valuable paradigm for global health management. One Health is an initiative that seeks greater integration of human and veterinary medicine in areas as diverse as infectious disease control and comparative and translational medical research. A major focus of One Health has been on infectious diseases shared by humans, production animals (e.g., cattle, sheep, pigs, and poultry), companion animals, and wildlife in the context of ecosystems and the physical environment. An area of One Health that has garnered much attention has been the emergence or reemergence of infectious disease, and the finding that ≈75% of newly reported human infections have emerged, and are therefore likely to continue to emerge, from an animal reservoir (*1*). Such zoonotic infections could be of the following types: infections transmitted directly from animals to humans; vector-borne infections in which an animal or human is infected by the vector; or infections in which animals act as a reservoir for disease transmission, including having the potential for contaminating human food and water sources.

A key goal of the evolving One Health paradigm includes surveillance of infectious diseases in domestic and wild animals to anticipate emergence of new zoonoses and protect humans. To achieve this goal, it is essential that global resources be allocated for more effective disease surveillance and reporting schemes that incorporate environmental, human, and veterinary health professionals. Many systems are in place nationally or globally to monitor human and production animal (and to a lesser extent, wild animal) disease (*2*), but major gaps in surveillance remain, particularly the lack of a surveillance infrastructure that includes companion animals.

From a One Health perspective, companion animals can serve as sources of zoonotic infections, as intermediate hosts between wildlife reservoirs and humans, or as sentinel or proxy species for emerging disease surveillance (*3*). The aims of this review are to define and quantify the role of companion animals in the human domestic and peridomestic environment, highlight the major companion animal zoonoses and the potential for emergence of new human infections transmitted from these species, emphasize the lack of global infectious disease surveillance in these species against the current background of human and production animal surveillance, and suggest how to address this major One Health deficiency in the future.

## Companion Animals

Companion animals have been domesticated by humans and kept primarily for social benefit (i.e., companionship, showing) or utilitarian purposes (i.e., hunting, military, or police activity; support for blind or deaf persons; guarding; and herding), They might be bred or wild caught with the intention of keeping them in the domestic environment. In some cultures, certain companion animal species also provide a food source. Companion animals encompass a spectrum that includes arthropods, caged birds, cats, chinchillas, dogs, ferrets, fish, guinea pigs, hamsters, horses, mice, poultry, rabbits, rats, and reptiles. This review focuses on the 2 major small companion animals: the domestic cat and dog.

Over millennia, cats and dogs have played an integral role in many aspects of human life. Human–companion animal interactions have a wide range of benefits to human health. These benefits include social development and improved quality of life associated with companionship, and inspiration afforded by pets (*4**,**5*). This bond with pets has strengthened over the past 50 years. The cat and dog have moved from the barn, into the house, and now, routinely, into the owner’s bed (*6*).

It is difficult to enumerate accurately the populations of pets kept in households in industrialized countries. Most recent estimates in the United States suggest that there are 72 million pet dogs kept by 37% of households and 81 million pet cats kept by 32% of households (www.avma.org/reference/marketstats/ownership.asp). In the United Kingdom, an estimated 8–10 million pet dogs live in 22%–31% of households, and the same estimated number of pet cats live in 18%–26% of households (*7*) (www.pfma.org.uk/pet-population-2011/).

The close association of humans with cats and dogs is not restricted to industrialized nations. The dog, in particular, has a major working and companionship role in many developing cultures, and shares the human environment in village life in many countries. The generally poor or nonexistent veterinary medical care afforded to these animals compounds the risk for transmission of infectious disease. More problematic is the vast number of free-roaming or community-owned dogs and cats that receive even less veterinary medical attention and provide a potential huge and uncontrolled reservoir for existing and new emerging zoonoses. Also of concern is the close association that domestic cats and dogs might have with wildlife, resulting in direct or indirect (e.g., through arthropod vectors) transfer of pathogens. This association might be particularly likely for free-roaming animals in rural areas, but it is also possible for pets in urban areas where there is potential for interchange of infectious agents between cats and dogs and wild animals such as raccoons, opossums, urban foxes, and wild rodent species.

## Zoonotic Infections in Companion Animals

There is a spectrum of infectious diseases of dogs and cats that are shared by humans (Table). Many of these are true zoonoses spread by direct contact between the species, and others are vector-transmitted (e.g., fleas, ticks, flies, and mosquitoes) diseases for which cats and dogs might act as reservoirs for the pathogen. Reverse zoonoses also occur in which disease is transmitted from the human reservoir to the dog or cat; the most contemporary examples are methicillin-resistant *Staphylococcus aureus* (*8*) and influenza A(H1N1)pdm09 virus (*9*) infections.

**Table:** Selected zoonotic infectious diseases of dogs and cats*

Route of human exposure	Agent	Principal clinical syndromes	Human surveillance mechanisms
Bites, scratches, or contact with exudates	*Bartonella* spp.† (bacteria)	Cats and dogs: subclinical fever, hyperglobulinemia, endocarditis, myocarditis, epistaxis, granulomatous rhinitis, uveitis, lymphadenopathy; humans: fever, malaise, endocarditis, myocarditis, meningitis, encephalopathy, lymphadenopathy, pulmonary granulomata, neuroretinitis, bacillary angiomatosis, bacillary peliosis	None
	*Capnocytophaga canimorsus* (bacterium)	Cats and dogs: subclinical oral carriage; humans: bacteremia	None
	*Francisella tularensis*‡ (bacterium)	Cats: septicemia, pneumonia; humans: ulceroglandular, oculoglandular, glandular, pneumonic or typhoidal (depending on route of inoculation)	NNDSS
	*Staphylococcus* spp.§ (methicillin resistant) (bacterium)	Cats, dogs, and humans: subclinical cutaneous infections, bacteremia	None
	*Yersinia pestis*‡ (bacterium)	Cats and humans: bubonic, bacteremic, or pneumonic (depending on route of inoculation and success of initial therapy)	IHR (pneumonic only), NNDSS
	Rabies (virus)	Cats, dogs, and humans: progressive CNS disease	NNDSS, WAHID
	Dermatophytes (fungi)	Cats and dogs: superficial dermatologic disease; humans: superficial dermatologic disease and deep tissue infections in immunocompromised patients	None
	*Sporothrix schenkii*‡ (fungus)	Cats and humans: draining cutaneous tracts	None
Contact with infected feces (ingestion unless otherwise indicated)	*Campylobacter jejuni and C. coli* (bacteria)	Cats, dogs, and humans: diarrhea and vomiting	WAHID, FoodNet
	*Escherichia coli* (bacterium)	Cats, dogs, and humans: diarrhea and vomiting	WAHID (STEC), NNDSS (STEC), FoodNet (STEC)
	*Salmonella* spp. (bacterium)	Cats, dogs, and humans: diarrhea and vomiting	WAHID, NNDS, FoodNet
	*Helicobacter* spp.¶ (bacterium)	Cats and dogs: vomiting; humans: reflux disease and vomiting	None
	*Yersinia enterocolitica* (bacterium)	Cats and dogs: subclinical infection or abdominal pain, vomiting and diarrhea; humans: diarrhea and vomiting	FoodNet
	*Entamoeba histolytica*# (ameba)	Dogs and humans: diarrhea and vomiting	None
	*Ancylostoma braziliense, Uncinaria stenocephala, A. caninum* (dog only) and *A. tubaeforme* (cat only)** (hookworms)	Cats and dogs: blood-loss anemia, diarrhea, unthrifty; humans: cutaneous larva migrans, eosinophilic enteritis	None
	*Baylisascaris procyonis* (roundworm)	Dogs: failure to thrive; humans: visceral larva migrans, CNS disease	None
	*Toxocara canis* (dogs) and *T. cati* (cats)†† (roundworms)	Cats and dogs: vomiting, diarrhea, failure to thrive; humans: ocular and visceral larva migrans	None
	*Strongyloides stercoralis*‡‡ (threadworm)	Cats and dogs: bloody diarrhea, blood-loss anemia, failure to thrive; humans: polysystemic disease	None
	*Echinococcus multilocularis* (cestode)	Cats and dogs: subclinical infection; humans: polysystemic hydatid disease	WAHID
	*Echinococcus granulosus* (cestode)	Dogs: subclinical infection; humans polysystemic disease	WAHID
	*Cryptosporidium* spp.§§ (coccidian)	Cats, dogs, and humans: diarrhea and vomiting	NNDSS, FoodNet, CryptoNet
	*Toxoplasma gondii*¶¶ (coccidian)	Cats: rarely diarrhea, polysystemic disease; dogs: neuromuscular and rarely polysystemic disease; humans: occular, CNS, and polysystemic disease	WAHID
	*Giardia* spp.## (flagellate)	Cats, dogs, and humans: diarrhea and vomiting	NNDSS
Contact with infected respiratory or ocular secretions	*Bordetella bronchiseptica* (bacterium)	Cats and dogs: sneezing and coughing; humans: pneumonia in immunosuppressed patients	None
	*Chlamydophila felis* (bacterium)	Cats: conjunctivitis, sneezing; humans: conjunctivitis	None
	F. tularensis*** (bacterium)	Cats: septicemia, pneumonia; humans: ulceroglandular, oculoglandular, glandular, pneumonic or typhoidal (depending on route of infection)	None
	*Streptococcus* group A (bacterium)	Cats and dogs: subclinical transient carrier; humans: strep throat, septicemia	None
	Y. pestis*** (bacterium)	Same as entry under bites, scratches, or contact with exudates	None
	Influenza A virus	Cats and dogs: respiratory disease, systemic disease; humans: respiratory disease	WHO Global Influenza Program
Contact with infected genital secretions	*Brucella canis*††† (bacterium)	Dogs: orchitis, epididymitis, abortion, stillbirth, vaginal discharge, uveitis, fever; humans: fever, arthralgia, headache, fatigue, myalgia, weight loss, arthritis/spondylitis, meningitis or focal organ involvement (endocarditis, orchitis/epididymitis, hepatomegaly, splenomegaly)	WAHID, NNDSS
	Coxiella burnetti*** (rickettsia)	Cats: subclinical, abortion or stillbirth; humans: fever, pneumonitis, lymphadenopathy, myalgia, arthritis	NNDSS
Contact with infected urine	*Leptospira* spp.(bacteria)	Dogs: fever, vomiting, pulmonary hemorrhage, renal and hepatic dysfunction, encephalopathy, uveitis; humans: fever, headache, myalgia, meningitis, pulmonary/hepatic and renal dysfunction, hemorrhagic complications	WAHID, some U.S. states
Flea-borne	*Bartonella* spp.† (bacterium)	Same as entry under bites, scratches, and contact with exudates	None
	*Y. pestis* (bacterium)	Same as entry under bites, scratches, and contact with exudates	None
	*Rickettsia felis* (rickettsia)	Cats: subclinical, fever; humans: fever, CNS disease	None
	*Rickettsia typhi* (rickettsia)	Cats: subclinical; humans: fever, polysystemic disease	None
Tick-borne‡‡‡	*Borrelia burgdorferi* (bacterium)	Dogs: subclinical infection, fever, polyarthritis, nephropathy; humans: polyarthropathy, cardiac and CNS disease	NNDSS
	*Anaplasma phagocytophilium* (rickettsia)	Cats and dogs: fever, polyarthritis; humans: fever, polysystemic disease	NNDSS
	*Ehrlichia* spp. (rickettsia)	Dogs: subclinical infection, fever, polysystemic disease; humans: fever, polysystemic disease	NNDSS
	*Rickettsia rickettsii* (rickettsia)	Dogs: subclinical infection, fever, polysystemic disease; humans: fever, polysystemic disease	NNDSS
Sandfly-borne	*Leishmania infantum* and *L. chagasi* (protozoa)	Cats and dogs: cutaneous lesions and polysystemic disease; humans: polysystemic (visceral) disease	None

*NNDSS, Nationally Notifiable Diseases Surveillance System; IHR, International Health Regulations (require report to WHO); Unthrifty, failure to grow or develop normally because of disease; CNS, central nervous system; WAHID, World Animal Health Information Database; STEC, Shiga-toxigenic *Escherichia coli*; WHO, World Health Organization. Further information on zoonoses of dogs and cats is available (www.cdc.gov/ncidod/diseases/list_zoonotic.htm, http://www.dpd.cdc.gov/dpdx, www.who.int/ctd/intpara, and www.who.int/topics/rabies/en/). †*B. henselae*, *B. koehlerae,* and *B. clarridgeiae* are transmitted among cats by *Ctenocephalides felis* fleas and *B. vinsonii* subsp. subsp. *berkhoffii*, *B. vinsonii* subsp. *aurpensis*, *B. washoensis*, *B. elizabethae*, *B. clarridgeiae,* and *B. quintana* are transmitted among dogs by *C. felis*, *C. canis,* and *Oropsylla montana* fleas and are also listed under flea-borne disease. There are other *Bartonella* spp. with zoonotic implications. Cats generally show development of a higher level of bacteremia than dogs and are epidemiologically linked more frequently to human disease. The vector is unknown for some *Bartonella* spp. ‡Dogs rarely shed enough organisms to be a public health risk. §Both *Staphylococcus aureus* and *S. pseudintermedius* have been implicated as zoonotic. ¶Most *Helicobacter* spp. found in cats and dogs are host-adapted species. When *H. pylori* is detected in a cat or dog, it is likely from reverse zoonotic transmission. #Infection of dogs in the United States is believed to be rare. **A. caninum is the species most commonly linked to enteritis in humans. ††Eggs require a larvation period in the environment to be infectious. Thus, direct transmission is less likely than exposure through environmental contamination. ‡‡Transmission is primarily by larval penetration of skin. §§Most humans are infected by *C. hominus* or *C. parvum*, and most cats and dogs are infected by *C. felis* or *C. canis*, respectively. These host-adapted species are less commonly identified in human feces, and information concerning disease associations with infection is limited. ¶¶Sporulation of oocysts occurs after passage into the environment; oocysts are environmentally resistant and it is estimated that only 1% of cats shed oocysts at any time. Thus, direct transmission is less likely than exposure through environmental contamination. ##Host-adapted and zoonotic assemblages exist. Cats and dogs can harbor zoonotic assemblages, but whether levels of infection result in reinfection of humans is not established. ***Can also be vector-borne. †††*Brucella* spp. transmission from dogs to humans is by direct or indirect exposure of organism to broken skin/mucous membranes by infected aborted fetuses, placental fluid, tissues, and semen. ‡‡‡*Borrelia* spp. DNA has been amplified from some ticks, but the extent of the role that ticks play in the transmission of these agents has not been fully ascertained.

Two companion animal zoonoses that exert a substantial effect globally on human health are rabies and zoonotic visceral leishmaniasis (ZVL). These are life-threatening human infections for which the domestic dog is the primary reservoir and for which control of the disease in the dog has the greatest benefit for human health (*10**,**11*).

## Human Zoonotic Disease Surveillance

Surveillance for human diseases that might originate with companion animals is not implemented uniformly across the globe. It ranges from statutory notifiable disease reporting to public access data mining (*12*).

The Global Public Health Intelligence Network (GPHIN), managed by the Public Health Agency of Canada and developed with the support from the World Health Organization (WHO), is a fee-based aggregator service for subscribers (www.phac-aspc.gc.ca/gphin/). Coverage ranges from infectious diseases to chemical exposures. GPHIN searches global media for relevant information and extracts content for review by analysts. Outbreaks identified by GPHIN feed into the Global Outbreak Alert and Response Network and the Global Early Warning and Response System, which bring together institutions such as the Food and Agriculture Organization, the World Organisation for Animal Health (OIE), and WHO and tap into a network of institutions and organizations available to provide resources and expertise in response to such alerts (www.who.int/csr/outbreaknetwork/en/). ProMed-mail, a program of the International Society for Infectious Diseases, is a similar data-searching resource (www.promedmail.org) that rapidly disseminates information.

A more quantifiable surveillance resource is available from the OIE World Animal Health Information Database (WAHID), which captures specified zoonotic human infections reported annually by member countries (http://web.oie.int/wahis/public.php?page=country_zoonoses). Rabies and leishmaniasis are reported into WAHID. Control of rabies, in particular, benefits from strong linkages between animal and human surveillance in industrialized countries. However, integrated surveillance for zoonoses is uncommon; only 19% of zoonoses surveillance systems cover humans and animals (*13*).

The Emerging Infections Program (www.cdc.gov/ncezid/dpei/eip/) at the Centers for Disease Control and Prevention coordinates population-based laboratory surveillance programs, some of which are relevant to transmission of zoonoses from companion animals. These programs include the Active Bacterial Core surveillance for invasive bacterial disease and the Foodborne Diseases Active Surveillance Network (FoodNet) for foodborne diseases caused by selected bacterial and parasitic pathogens. Surveillance of influenza in humans occurs at global, regional, and national levels. For example, WHO coordinates a network of 111 national influenza centers in 83 countries, which are supported by 4 regional reference laboratories (*12*).

## Surveillance of Zoonoses in Production and Wild Animals

Global surveillance of infectious diseases, including zoonoses, in production animals is coordinated by OIE through the veterinary medical infrastructure of the 178 member nations. The disease surveillance system of each country is based in the governmental veterinary service that collects and disseminates disease surveillance findings. In addition to this official infrastructure, there is a major role for private veterinary practitioners and paraprofessionals who communicate directly with producers and the general public.

OIE member nations must disclose relevant information about animal diseases, and OIE is obliged to make immediate reports to governments on emerging diseases and other major epidemiologic events. Members must report occurrence of animal diseases considered by OIE to have major socioeconomic or public health roles, emergence of new diseases, and major epidemiologic events within 24 hours of the event. OIE publishes and disseminates monthly reports on the global animal disease situation. The capacity of OIE to relay information about the global animal disease situation is based in WAHID, but OIE also reports unofficial (but reliable) information of global health concern.

Recently, OIE has expanded surveillance of wildlife disease by collating data from member countries and providing an overview of disease events and pathogens in these species. Data collection through WAHID enhances dissemination of this information.

## Surveillance of Zoonoses in Small Companion Animals

In comparison with human and livestock diseases, none of the international health agencies are mandated to coordinate surveillance of diseases in companion animals. In addition, surveillance systems exist for only a few of the small animal zoonoses (e.g., rabies and leishmaniasis).

### Rabies

An estimated 55,000 persons die each year of rabies, primarily because of dog bites and subsequent infection with canine rabies, in resource-poor areas of Africa and Asia. The effects of rabies are considerably less in industrialized countries, which might be attributed to widespread compliance with mandatory dog leash laws, vaccination, and resulting high vaccination coverage.

In developing nations, problems relate to surveillance and control, and there is a lack of diagnostic capacity, misdiagnosis, poor incentivization, difficulties of communication (within and between sectors), and logistical and financial constraints. Despite being one of the most serious and frequently fatal zoonoses, rabies is still underreported throughout the developing world, and 100-fold underreporting of human rabies is estimated for most of Africa (*13*). For human cases, underreporting arises for numerous complex reasons, but studies from Tanzania indicate that proxy measures (e.g., the incidence of bite injuries from suspected rabid animals from hospitals and bite clinics) can provide a valuable source of surveillance data (*13*). New initiatives involving mobile telephone technologies are being evaluated that aim to address deficits in communication and facilitate more effective responses, e.g., by providing real-time access to rabid dog-bite injury data from hospital clinics (*14*).

### Zoonotic Visceral Leishmaniasis

ZVL is another example of a major human infectious disease in which surveillance and control of the infection in the domestic dog population is fundamental. ZVL is a chronic and potentially lethal human disease caused by the protozoan parasite *Leishmania infantum* in the Old World and *L. chagasi* in the Americas. These agents are transmitted by the bite of an infected sandfly. ZVL involves a canid (e.g., domestic dog or fox) reservoir of the parasites. This disease is endemic to the Mediterranean basin, Middle East, and South America and is emerging within non–disease-endemic areas because of transportation of dogs from disease-endemic areas, expansion of the geographic range of free-ranging wildlife reservoir species and sandfly vectors because of climate change, and human incursion into previously uninhabited wildlife ecologic niches (*15*).

Visceral leishmaniasis (VL), including ZVL and anthroponotic visceral leishmaniasis caused by *L. donovani*, is estimated to affect 12 million persons worldwide, and 350 million persons are at risk for infection. Each year, there are an estimated 500,000 new human cases of VL (*16*). A database summarizing published literature on the spatial distribution of VL is available (http://apps.who.int/tools/geoserver/www/ecomp/index.html).

Measures established for the global surveillance and control of this major infectious disease have been reviewed (*15*). Surveillance of infection in the canine reservoir is limited and control measures are practiced variably in different countries. A coordinated One Health approach is essential for the effective surveillance and control of this disease.

## Opportunities for Small Companion Animal Infectious Disease Surveillance

### Influenza

Evidence from recent influenza outbreaks suggest that cats might be infected more frequently with influenza viruses than previously considered. Following reports of fatal highly pathogenic avian influenza (HPAI) virus (H5N1) infection in domestic cats and zoo felids fed virus-infected chickens (*17*), laboratory studies confirmed that HPAI virus (H5N1) can infect domestic cats and cause severe multisystemic disease (*18*). In addition, field observations indicated that HPAI virus (H5N1) infection might cause widespread deaths in cats (*19*).

The implications of these findings are that, during HPAI virus (H5N1) outbreaks, domestic cats are at risk for disease or death from HPAI virus (H5N1) infection, and that unusual deaths of cats in areas at risk for HPAI virus (H5N1) infection might act as a warning signal for presence of the virus. Domestic cats might also have a role in the epidemiology of HPAI virus (H5N1) infection in poultry, humans, and other species. Surveillance in HPAI virus (H5N1)–endemic areas should include felids and other carnivores showing unusual illness or deaths (*17*).

During influenza A(H1N1)pdm09, multiple instances of companion animal (ferret, cat, or dog) infection with influenza A(H1N1)pdm 09 virus were confirmed by laboratory testing (*9*). Transmission in these cases was from ill owners to their pets. Experimental studies showed that this virus caused severe pneumonia in cats, but not extrarespiratory disease (*20*).

In 2004, equine influenza virus (H3N8) was transmitted to racing dogs in Florida, USA (*21*). Subsequently, this virus has become enzootic in kennels and shelters in major US cities (*22*). Although infections are uncommon in domestic pets, the circulation and properties of this virus in dogs should be monitored to detect changes in the viral host range.

Although the risk for influenza transmission from companion animals to humans is assumed to be low, dogs have a role in the evolution of influenza viruses (*23**–**25*). Because of the constantly evolving ecology of influenza viruses, including interspecies transmission, surveillance for novel influenza viruses at the pet–human interface is well justified and should be encouraged.

### Other Emerging Infectious Diseases

The public health role of many zoonotic agents of small companion animals is underrecognized or poorly understood by medical and veterinary health care providers and pet owners. Furthermore, small companion animals might play a major role in potential zoonoses of the future, either by acting as a reservoir or as an intermediate host. Information about known and potential zoonoses associated with cats and dogs is emerging rapidly and needs to be more efficiently disseminated. Two examples of emerging zoonoses are the arthropod-transmitted rickettsioses and bartonellosis (Table). The prevalence of these agents in cats, dogs, fleas, and ticks has been reported from several countries, and groups such as the Canine Vector Borne Disease Forum (www.cvbd.org/) collate and disseminate relevant information.

Discussion of vector-borne diseases highlights the ability of cats and dogs to act as proxy species for emergence of disease in the human population. For example, dogs might act as a proxy species for surveillance of borreliosis (Lyme disease), and dogs have been used effectively as proxies for risk for human spotted fever rickettsioses (e.g., Rocky Mountain spotted fever caused by *Rickettsia rickettsii*) in newly emergent areas (*26*). In addition, serologic surveillance for *R. typhi* or *R. felis* in cats might be useful for predicting areas of human risk for endemic typhus (*27*).

The science of surveillance using animal proxies is relatively underdeveloped and constrained by the lack of alignment and communication between the human health sector and the veterinary medical sector and lack of infrastructure or quantitative methods for linking animal and human health data (*28**–**31*). However, companion animal proxies offer a clear opportunity to address some of the deficits that are currently faced in the field of zoonosis surveillance.

## Existing Schemes

Although global surveillance of infectious diseases in humans and production animals is relatively well developed, with the exception of surveillance of rabies and leishmaniasis, efforts to instigate such surveillance for other canine and feline infectious diseases have been minimal. Most surveillance in these species has been conducted at a national or regional level as part of specific and finite research programs without standardization of nomenclature, disease definition, or means of diagnosis.

A good example of a successful national surveillance investigation is that reported by the Companion Animal Parasite Council in the United States for distribution of the major canine tick-borne infections, intestinal parasites, and heartworm disease (www.capcvet.org/maps/index.html). In the United Kingdom (http://uk.cicadasurvey.com/) and Australia (www.diseasewatchdog.org/index.php/login), 2 veterinary vaccine manufacturers have introduced simple sentinel practice-based reporting schemes for canine and feline infectious diseases. These data provide proof-of-principle for the sentinel practice concept. In the United Kingdom, the Small Animal Veterinary Surveillance Network scheme has shown the feasibility of using shared practice record-keeping software to enable veterinarians to input data related to disease diagnoses made in their practices (*32*). Data-mining from corporate small animal practices with shared computer databases has been successfully undertaken in North America through the Banfield Hospital group (*33*). Combining these local efforts on a global scale will improve the data obtained and enable a surveillance system that is currently missing.

## Recommendations for the Future

This review has highlighted the potential for transmission of infectious disease from cats and dogs to humans. Given the close proximity to humans and the role of companion animals as a source of zoonoses, the absence of these species from international agendas for One Health represents a gap that requires urgent attention.

To highlight the issues surrounding this area, the World Small Animal Veterinary Association (WSAVA) has recently formed a One Health Committee, and the present position paper has arisen from discussions held by this committee. The WSAVA One Health Committee draws together expertise in companion animal infectious disease research, and 1 goal of this committee is to enhance understanding of the infrastructure required for effective integrated global infectious disease surveillance (www.wsava.org).

The establishment of an effective global companion animal infectious disease surveillance network presents a major political, financial, and scientific challenge. We present a proposal by which such a system might develop. Once a current and potentially emergent infectious disease had been identified, the first issue is to define that disease by using internationally standardized nomenclature and to develop standardized laboratory diagnostic procedures to confirm the presence of infection. Such diagnostic tests would ideally be highly sensitive and specific, simple, affordable, robust, and designed for practice-based use. In parallel would be the requirement for development of a computerized database and the centralization, ownership, management, and data-mining that must occur with such a resource.

Once this infrastructure is in place, the next challenge would be to capture high-quality field data from across the globe. With a simple computer-based or mobile telephone–based reporting system and the availability of appropriate diagnostics, it would be feasible for a sentinel small animal veterinary practice to collect and input local field data. This process would require major educational input to veterinary practitioners and their support staff, and instruction on the need and role of their involvement in a global One Health alliance, in addition to the practicalities of data entry. An organization such as WSAVA, which currently represents some 160,000 front-line small animal veterinarians in 80 countries, would be ideally placed to act as a point of contact for initiation of such a scheme.

Assuming that such a scheme could generate reliable global infectious disease surveillance data, this information would then need to be disseminated to regulatory authorities and to human and veterinary public health professionals. The information might be web-based and comprise real-time disease distribution maps; global disease alerts; and fundamental information on the epidemiology, transmission, pathogenesis, diagnosis, and management of highlighted infections. The education of human and veterinary medical personnel will be a crucial step in this process.

Although the desirability of a global One Health infectious disease surveillance system that includes small companion animals should now be clear, the hurdles to be crossed to establish such a scheme are challenging. A fundamental issue becomes who will assume the leadership responsibility to plan for this much needed system? Should the funding for disease surveillance be the responsibility of local governments or nongovernmental organizations (given their role in animal and human health), or should responsibility be placed with the pet owner (who elects to keep a companion animal) or the industry (i.e., pet food and pharmaceutical) that generates income from the keeping of pet animals or breeders that raise and sell these animals? Although a plan for pet owners and industry to take responsibility might be feasible in more affluent industrialized nations, it would be unlikely to be effective in developing nations. Alternatively, a cost-sharing model might be considered in which surveillance becomes a public–private partnership. These are not simple questions, but the first stage in this process is recognition of the problem by those in authority and the brokering of discussions between interested parties. We hope that this report might define historical and evolving facts and thereby prompt such discussions in the near future.
